# Blood pressure management protocol based on transtheoretical model effectiveness on self- care: A systematic review

**DOI:** 10.34172/hpp.42814

**Published:** 2024-10-31

**Authors:** Habibeh Barzegar, Sheida Sodagar, Mohammadreza Seirafi, Mostafa Farahbakhsh, Touraj Hashemi

**Affiliations:** ^1^Department of Health Psychology, Karaj Branch, Islamic Azad University, Karaj, Iran; ^2^Research Center of Psychiatry and Behavioral Sciences, Tabriz University of Medical Sciences, Tabriz, Iran; ^3^Department of Psychology, Faculty of Education and Psychology, University of Tabriz, Tabriz, Iran

**Keywords:** Hypertension, Self-care, Self-management, Systematic review, Transtheoretical model

## Abstract

**Background:**

Hypertension is a prevalent medical condition associated with cardiovascular and kidney diseases, leading to premature death and disability. Despite medication use, hypertension continues to rise due to unhealthy lifestyles. Self-care strategies play crucial roles in effectively treating hypertension. We aimed to evaluate the effectiveness of interventions based on the transtheoretical model (TTM) in improving self-care, self-efficacy, and health-related quality of life (HRQOL) in hypertensive adults.

**Methods:**

A comprehensive search was performed in multiple databases using appropriate search strategies. Two independent reviewers screened articles, and assessed their adherence to the inclusion and exclusion criteria. The risk of bias in randomized controlled trials was assessed by the Cochrane Collaboration tool and quasi-experimental studies using the Joanna Briggs Institute (JBI) Critical Appraisal Checklist for Quasi-Experimental Studies.

**Results:**

The review included 24 studies with a total of 6553 participants. Most interventions aimed to encourage a healthier lifestyle and improve diet and physical activity. The message was delivered through various methods such as slide/tape messages, individual education/counseling sessions, telephone-delivered interventions, and multimedia training software based on the TTM. Control groups received physician visits or attended lifestyle lectures but did not receive specific behavior change programs like the intervention groups.

**Conclusion:**

Our results indicated that the TTM and stage of change model can be an appropriate framework for delivering educational messages to patients.

## Introduction

 Hypertension, which is characterized by systolic blood pressure (SBP) ≥ 140 mm Hg and/or diastolic blood pressure (DBP) ≥ 90 mm Hg, has a significant impact on millions of individuals worldwide.^1^ The implementation of lower thresholds for diagnosis has resulted in a higher prevalence of hypertension than ever before.^2^ This medical condition is closely associated with various cardiovascular and chronic kidney diseases, such as heart failure and myocardial infarction,^3^ therefore it is crucial to note that hypertension stands as the foremost preventable cause of premature death and disability.^4^

 To effectively treat hypertension, patients must adhere to self-management guidelines and collaborate with their healthcare provider^5^ Some effective self-management strategies include increasing awareness and education, medication adherence, stress management, lifestyle improvements, and regular follow-ups with healthcare professionals.^6,7^ barriers to hypertension self-management include limited knowledge about hypertension, other health problems, and improper access to community resources. While support from a family member is known as a facilitator.^8^

 Two additional barriers to reaching sufficient care and management in chronic disease are a lack of motivation and a disability in problem-solving. These problems can be overcome through self-efficiency and balanced decision-making, which in turn lead to increased patient involvement. Self-efficacy is a key factor in making lifestyle changes that can help improve chronic conditions like hypertension. Individuals with high self-efficacy strongly believe in their ability to make specific changes to improve their health. Conversely, individuals with low self-efficacy lack confidence in their ability to make positive changes in their health behaviors.^9^ The transtheoretical model (TTM) is a useful framework for addressing these issues and gaining a better understanding of health behaviors.

 The core constructs of TTM include stages of change, processes of change, decisional balance, self-efficacy, temptation, and critical assumptions. Temptation reflects the intensity of urges to engage in a specific behavior when in the midst of difficult situations. Prochaska and Velicer in 1997 outline the list of seven assumptions that drive transtheoretical theory, research, and practice: (1) No single theory can account for all of the complexities of behavior change. (2) Behavior change progresses over time through a sequence of stages. (3) Stages are open and stable to change just as chronic problem behavior factors are both stable and open to change. (4) Without planned interventions, people will remain caught in the early stages because there is no inherent motivation to progress through stages of intentional change as there seems to be in stages of physical and psychological development. (5) The majority of at-risk populations are not ready for action and will not be served by traditional action-oriented prevention programs. (6) Specific processes and principles of change need to be applied to specific stages for proper progress through the stages. (7) Chronic behavior patterns are often under some combination of biological, social, and self-control. Stage-matched interventions are primarily designed to increase self-control. Processes of change refer to the covert and overt events that people utilize to progress through the stages of change. Decisional balance demonstrates the individual’s evaluation of the pros and cons of making a change.

 Considering the high prevalence of hypertension and its associated health conditions, effective management is crucial to reduce its morbidity and mortality. Further, self-care practices including lifestyle modifications, medication adherence, and regular monitoring are essential for individuals with hypertension. TTM can provides a framework for understanding how individuals change their behavior over time. Hence, application of TTM to blood pressure management could enhance self-care strategies.

 While the TTM has been widely studied in various health behaviors, there may be limited research specifically focusing on its application in blood pressure management and self-care. Understanding how TTM can be effectively integrated into hypertension management protocols is crucial. Previous studies may yield inconsistent results regarding the effectiveness of TTM-based interventions for hypertension self-care.

 In this study, a systematic review is performed to examine blood pressure management protocols based on the TTM and evaluate their role in measures including self-care, self-efficacy, and quality of life.

## Method

###  Protocol and registration

 This Synthesis without Meta-analysis (SWiM) is developed using the Preferred Reporting Items for Systematic Reviews and Meta-Analyses (PRISMA) guideline checklist.^10^ This review has been registered in the Prospective Register of Systematic Reviews (PROSPERO) with registration number CRD42023412499.

###  Search strategy

 To find all relevant studies, a systematic search was conducted in the following databases: PubMed, Scopus, Embase, Web of Science, APA PsycNET, and the Cochrane Central Register of Controlled Trials (CENTRAL) from inception up to July 2023 in English. The search was then updated in July 2024. The search strategies were developed using both medical subject headings (MeSH) and free-text terms which contained the following main terms: ‘hypertension’, ‘high blood pressure’, ‘elevated blood pressure’, ‘hypertensive disease’, ‘transtheoretical model’, ‘stage of change’, ‘behavior change’, ‘change stages’, ‘self-care’, ‘self-care behavior’, ‘self-management’, ‘self-efficacy’, ‘health-related quality of life’. Vocabulary and syntax were adjusted across databases.

 The reference lists of retrieved articles were also reviewed for potentially eligible publications, and previous review articles were checked for additional references. The results of the searches were uploaded to an EndNote library (v. 20.4.1)

###  Selection/screening of studies

 To enhance the credibility and robustness of the study, the identification of the studies, including the search and the selection process was conducted by two independent researchers with formal degrees in health sciences and a strong background in conducting systematic reviews. They were also highly skilled in database searching and documenting search processes and results. They independently screened all articles using Rayyan — a web and mobile app for systematic reviews (2016) 5:210.^11^ Abstracts and article helpfulness were assessed, and duplicates were eliminated. Then, inclusion and exclusion criteria were applied. Researchers were blinded to each other. Any discrepancies were resolved by a third reviewer.

###  Eligibility criteria

 Eligibility criteria are defined as all study participants must be adults (over 18 years old) and have hypertension (as diagnosed using any recognized diagnostic criteria). Articles were restricted to the English language. Interventions had to be based on the TTM and have to consider their effectiveness in either self-care, self-efficacy, or health-related quality of life (HRQOL). Books, book chapters, and conference abstracts were excluded.

###  Data extraction

 Two independent authors extracted important data from included articles into a Microsoft Excel (Microsoft Corporation. (2018). Microsoft Excel. Retrieved from https://office.microsoft.com/excel) sheet which was designed based on the Cochrane Data Extraction and Assessment Form. Any disagreement was discussed with a third author. In case of missing data, the authors were contacted. Essential studies’ characteristics were extracted, including bibliographic information (authors, year of publication, origin), study design, population characteristics (number, age, and gender), type of comparison, and outcomes related to study objectives including self-care, self-efficacy, and HRQOL. The type and duration of intervention, type of the studied behaviors, behavior change, and change in blood pressure were also extracted.

###  Risk of bias assessment

 The quality of randomized controlled trials was assessed using the Cochrane Collaboration tool which evaluates biases related to random sequence generation, allocation concealment, blinding of participants and personnel, blinding of outcome assessment, incomplete outcome data, selective reporting, and other biases. Risk of bias assessments for quasi-experimental studies were conducted using the Joanna Briggs Institute (JBI) Critical Appraisal Checklist for Quasi-Experimental Studies (Supplementary file 1). This checklist consists of 9 questions regarding the methodology and reporting of the research items. Studies were rated as high, moderate, and high quality if they were subjected to 0-1, 2-3, or more than 3 No/Unclear answers. One reviewer independently assessed the risk of bias for each study, with verification by another. If needed, disagreements were resolved by a third reviewer.

###  Data synthesis

 Due to the great diversity in the methods and results of the studies, as well as the high heterogeneity of the results of the studies, meta-analysis was omitted in this study and the results were reported in a narrative form.

## Results

**Figure 1:**
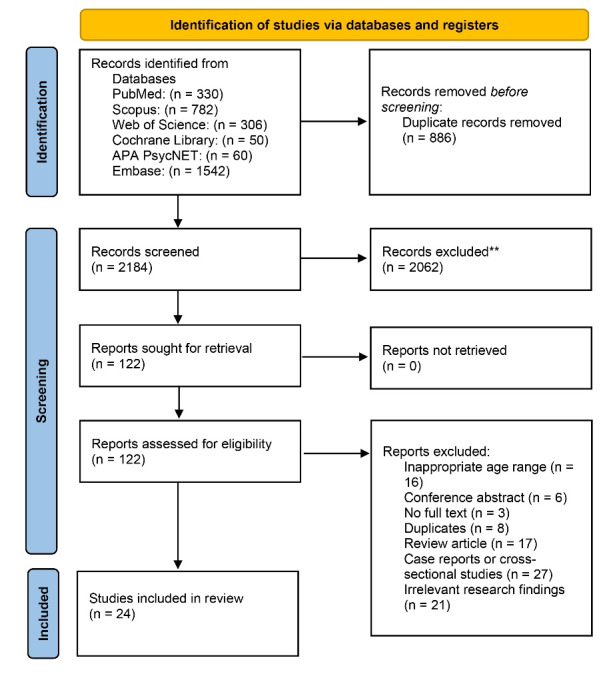
The PRISMA flow diagram presenting the search results and selection process

 The comprehensive systematic search identified 3070 studies. After the exclusion of duplicates, 2184 studies were screened. After excluding articles due to obviously irrelevant topics/abstracts, 122 articles were identified as potentially eligible. The full-text assessment was performed on the remaining papers, and 98 of them were excluded for failing to meet the eligibility criteria. Finally, a total of 24 studies were included in the review. In Figure 1 the PRISMA flow diagram summarizes the selection process.

Table 1 show the interventional information of the included studies, respectively. Twenty-four studies were included, which assessed the outcomes of self-care, self-efficacy, and HRQOL in hypertensive patients. Among the studies included in this review, 18 were randomized controlled trials, with one of them being a clustered randomized controlled trial, one study appeared to meet eligibility criteria for this review^12^ but was excluded for only discussing the rationale, design, and recruitment rather than the actual outcomes. The remaining studies were designed as pretest-posttest quasi-experimental, nonequivalent pretest-posttest comparison group, and one-group repeated measures design. The publication date of studies ranged from 1989 to 2022, while approximately 80% were published in 2010 and later. Studies involved 6553 participants. Sample sizes ranged from 37 the least to 1227 the most. Participants were all adults,18 and older, and the mean age for the most senior age group was 65 ± 11. Female participant’s percentages ranged from 1.1% to 86.0%. Eleven studies were conducted in the USA,^13-23^ three in Iran,^9,24,25^ two in Brazil,^26,27^ the United Kingdom,^28^ and the rest were performed in Australia,^29^ Japan, Bangladesh,^30^ Malaysia,^31^ Canada,^32^ China,^33^ and the Netherlands.^34^ In most studies, participants were randomized to intervention and control groups, but in six studies, participants were divided into three groups.^13,17,20-23^

###  Interventions, type, and duration

 Most reviewed studies aimed to encourage participants to achieve a healthier lifestyle and improve their diet, physical activity, etc. The message was delivered in different ways. In one study, subjects were shown slide/tape messages and asked to complete a questionnaire about their future blood pressure control plans.^13^ Another way to deliver the message was by emphasizing the costs, beneﬁts, barriers to a healthy lifestyle, goal setting, time management, and social support.^29^ In several studies, given intervention was matched with the stage of change classiﬁcation obtained at baseline, including individual stage-speciﬁc education/counseling session^14^ or telephone-delivered, transtheoretical stage-matched intervention.^20,23^ Other studies do not emphasize giving stage-matched intervention but perform an assessment of behavior change. In the study by Dickman et al, the clinic visit consisted of three parts: checking vital signs and concerns, Interactive education, and goal setting.^15^ Also, in many of them, the message was delivered in a session for an hour to 2.5 hours. On the other hand, control groups also received physician visits or attended lectures about lifestyle but didn’t receive specific behavior change programs as for the intervention groups.^16,26^ In one study, participants received in-person health education, a health education booklet, and SMS text messaging to develop awareness and knowledge and motivate them for behavior changes.^30^ Multimedia training software, a Pro-Change Program for High Blood Pressure Medication, highly tailored and interactive text, and voice recognition messages, all based on the TTM model, were other ways of encouraging participants.^18,24,28^ In the PREMIER trial^21,22^ included lifestyle recommendations plus DASH” intervention included the same established guidelines plus the Dietary Approaches to Stop Hypertension (DASH) dietary pattern. Riches et al performed the SaltSwap intervention to reduce dietary salt intake by encouraging individuals to swap to lower-salt alternatives.^35^ Duration of the intervention varied from 3 to 18 months, and in most of the studies, evaluations were conducted before, during, and after the intervention to obtain correct assessment.

###  Quality assessment 

**Figure 2:**
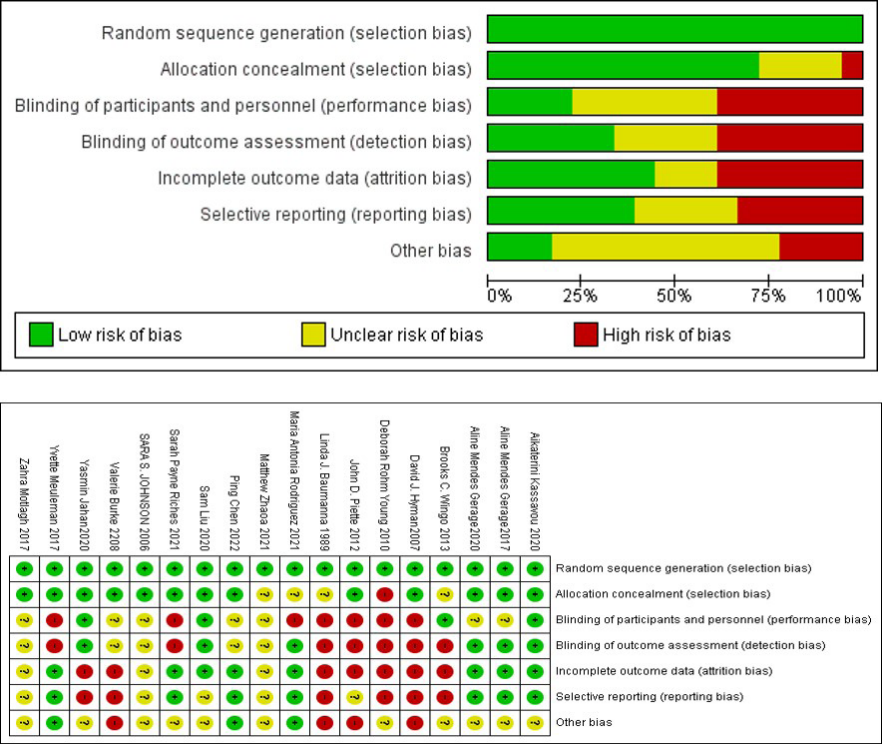
Risk of bias graph and summary

 As previously mentioned, randomized controlled trials were assessed by the Cochrane Collaboration tool to assess the risk of bias for randomized controlled trials. Of 18 randomized controlled trials, nine were high quality, five were moderate, and four were low quality. Figures 2A, and B demonstrate the risk of bias graph and summary for randomized controlled trials studies. As shown in Table 2, the quality of the two studies was rated as high (Jalali et al,^4^ Naeemi et al^4^) and the rest of the studies had moderate quality. The two studies with high quality had a control group.

###  Change in blood pressure

 Although nearly all studies had baseline blood pressure measurements, sevenhave performed blood pressure assessments post-intervention and reported blood pressure change. Dickman et al reported a 30 mm Hg reduction in SBP.^15^ Fort et al reported that those participants attending more healthy lifestyle education sessions, a more significant decrease in systolic (Coefficient: −9.14; *P* < 0.001) and diastolic (Coefficient: −3.72; *P* = 0.002) blood pressure was observed.^16^ In the VAMOS study, the intervention led to a significant reduction of brachial SBP and central SBP and brachial DBP (131.3 to 125.1; 123.6 to 119.0; 123.6 to 119.0 mm Hg ). However, no significant changes were observed in body composition, heart rate, and arterial stiffness parameters in both groups.^26^

 SBP and DBP (mm Hg ) were markedly more significant in the intervention group in the study done by Jahan et al.^30^

 In the study conducted by Karupaiah et al, intervention group patients had higher mean SBP (141.6 ± 15.9 vs. 131.6 ± 14.7 mm Hg ) and DBP (88.7 ± 10.3 vs. 82.7 ± 9.5 mm Hg ) compared to standard therapy patients at baseline, demonstrating greater degree of uncontrolled hypertension. Assessments after intervention indicated that the modified therapy, compared to the standard therapy group, significantly reduced SBP (−12.1 ± 17.6 vs. −5.7 ± 14.9 mm Hg, *P* = 0.022). However, the decrease in DBP (−7.7 ± 121.6 vs. −4.2 ± 11.2 mm Hg, *P* > 0.05) was insignificant between groups.^31^ Piette et al and Meuleman et al also stated a significant reduction in blood pressure in intervention groups.^19,34^

###  Behavior change 

 Participants were encouraged to make positive improvements in their lifestyle. Increasing physical activity, diet changes, medication adherence, weight loss, smoking cessation, blood pressure monitoring, and health care utilization are some examples of improved behavior changes in the reviewed studies. Improvements in the diet included the following items:

- Reduction in fat consumption which has been shown in three studies^22,26,27^
- Using less salt or being described as having lower sodium intake has been investigated in six studies^13,17,22,30-32^ and has been proven to have a direct effect on hypertension. For instance, Jahan et al^30^ reported salt intake less than 6 g/d, and showed significant chronological improvement (*P* < 0.001).
- Restricting sugar intake which was only being evaluated in Gerage et al study,^27^
- Increasing fruit and vegetable intake as described in four studies.^27,30-32^

Increasing physical activity was reported in 16 studies. Baumann et al assessed changes in physical activity by using stairs more.^13^ Burke et al assessed Physical activity self-efﬁcacy, introducing self-efficacy as a mediator of behavior change. In their study, women had a better response with usual care; hence, they concluded that the gender of the participants was a moderator of response to the intervention.^29^ Moreover, Dickman et al recorded an 86 min/wk increase for exercise having a 130 min/wk baseline. In the study conducted in Costa Rica and Chiapas, the percentage of participants who met the recommended minutes of physical activity per week. However, no significant change was observed for physical activity in intervention vs. comparison group participants.^16^ In the VAMOS study,^27^ participants in the control group increased the total daily time spent in sedentary behavior (F = 4.61; *P* = 0.04) and showed decreases in total physical activity from pre-intervention to post-intervention (F = 4.61; *P* = 0.04). Karupaiah et al evaluated pedometer steps (counts per day) and total activity (MET, min/wk) which indicated significant increase from baseline to 6 months.^31^

 Wingo et al^21^ and Baumann et al^13^ recorded weight loss among participants. Body weight monitoring was also recorded in a trial performed in a Rural Community in Bangladesh.^30^ Increase in medication adherence was observed in six studies.^18-20,23,28,33^ Two studies stated positive changes to restrict smoking.^13,17^ Self-care behaviors also improved significantly. Naeemi et al showed that the proportion of hypertensive self-care behaviors before educational intervention in the experimental and control groups. The mean self-care score in intervention and control groups changed from 60.02 to 79.4 and 59.1 to 59.7, respectively. However, this change was not statistically significant (*P* > 0.05).^25^

###  Self-efficacy

 Self-efficacy was investigated in five studies.^14,16,24,27,29^ Overall, based on the findings of these studies, self-efficacy played a significant role in facilitating behavioral changes, particularly in the context of dietary change and physical activity. Burke et al investigated moderators and mediators of behavior change in a lifestyle program that led to introducing self-efﬁcacy as a mediator of dietary change post-intervention (effect size [ES] 20.055, 95% conﬁdence interval [CI] 20.125, 20.005) and at follow-up (ES 0.054, 95% CI 20.127, 20.005), and in physical activity post-intervention (ES 0.059, 95% CI 0.003, 0.147). Daley et al^14^ executed a one-group repeated design study. They evaluated exercise self-efﬁcacy measured using the 13-item McAuley Exercise Self-Efﬁcacy scale.14 and was rated on a scale of 0%, indicating not at all conﬁdent to 100%, indicating highly conﬁdent. According to their results, exercise self-efficacy and benefits increased after the intervention and 70% of participants increased exercise performance. In contrast, in the study conducted in Mexico,^16^ no significant change was observed in self-efficacy.

 In the VAMOS study,^27^ self-efﬁcacy was evaluated by the self-efﬁcacy for exercise and eating habits scale. The scales contained items that measured the level of conﬁdence to perform and maintain an exercise and healthy eating habits routine. In the controlled study by Jalali et al, the intervention group significantly increased physical activity behavior self-efficacy.^24^ According to their results, a significant increase in physical activity in both groups, yet this increase was significantly higher in the intervention group compared to the control group (36.02 to 146.16 and 33.41 to 54.41, respectively, *P* < 0.001).

###  Health-related quality of life

 Three studies investigated HRQOL and reported mixed findings; two of them showed positive changes in HRQOL and one showed no significant changes.^22,27,28^ More specifically, in the study by Young et al,^22^ the intervention was defined as increasing physical activity to a minimum of 180 min per week, decreasing sodium intake to ≤ 2300 mg/d, and decreasing total fat intake to ≤ 30% and saturated fat to ≤ 10% of calories per day. They showed that dietary change, mainly percent of daily caloric intake in total fat and saturated fat, and fruits and vegetables intake, was significantly associated with differences in HRQOL scores. An increase in one daily serving of fruits and vegetables leads to increased HRQOL scores of magnitudes between 0.21 and 0.25 units.

 In the trial performed by Gerage et al the quality of life was evaluated by the general question of the World Health Organization Quality of Life questionnaire, brief version: “Considering the last two weeks, how do you evaluate your quality of life?” The answer options were “very bad,” “bad,” “neither bad nor good,” “good,” and “very good.” The trial analyzed the percentage of participants with good or very good quality of life in pre- and post-intervention groups. It was demonstrated that the intervention, the VAMOS program, improved HRQOL (44% vs 92%; *P* < 0.05) in patients with hypertension. In contrast, no statistically significant differences were found in the quality of life between groups in a study performed by Kassavou et al.^28^

**Table 1:** Interventional information of the included studies

**Author, year**	**Country**	**Intervention (s)**	**Sample size**	**Sample age range**	**Duration of intervention / follow-up**	**Target behavior**	**Overall results **
Baumann et al,^13^ 1989	USA	Three slide/tape messages (Standard, Action plan, Wellness thinking) After viewing the slide/tape message, subjects completed a two-page questionnaire covering their plans for future blood pressure screening, intentions to improve health habits, knowledge and concern about high blood pressure and its treatment, and symptoms they believed to be associated with high blood pressure.	296	17-65	Nine months	Intention to: 1) Use stairs more PA 2) Use less salt 3) Trim fat off meat more (reduction in fat consumption) 4) Lose weight 5) Smoke less	The interventions resulted in higher reported intentions and behavior change up to 9 months later, although few differences were statistically significant. Hence, hypertension education should include information on blood pressure-lowering strategies..
Burke et al,^29^ 2008	Australia	Participants were encouraged to achieve a low-sodium, low-fat diet rich in fruit and vegetables.Increased ﬁsh consumption and increased physical activity. The program emphasized barriers, costs, and beneﬁts of a healthy lifestyle, goal setting, time management, and social support.	241	40-70	Four months (1 year follow-up)	1) Diet self-efﬁcacy 2) PA 3) Reduction in saturated fat consumption 4) Change in time spent in exercise	Sex was a mediator of response to diet and physical activity post-intervention, with women receiving standard care and males participating in the program exhibiting higher levels of response. Also, change in self-efficacy was a mediator of dietary change and in physical activity.
Daley et al,^14^ 2009	USA	Based on the exercise stage of change classiﬁcation obtained at baseline, women were given a stage-speciﬁc (stage-matched) intervention that consisted of (1) a 2.5-hour face-to-face.Individual stage-speciﬁc education/ counseling session, (2) performance of a moderate-vigorous aerobic exercise prescription on their own, (3) 3 weekly follow-up phone calls to review the exercise diary (Weeks 1–3), (4) a ﬁnal face-to-face visit (Week 4), and (5) a ﬁnal phone call as follow-up to assess whether the women felt conﬁdent that they could continue the established exercise plan (Week 5).	40	18-55	Six or more months	Exercise self-efﬁcacy	Following the intervention, the majority of women—85%—went to or stayed in the highest levels of readiness—the action or maintenance phases of change; none of them experienced a relapse. Seventy percent of individuals improved their exercise performance, and exercise self-efficacy and benefits rose while obstacles reduced.
Dickman et al,^15^ 2012	USA	Shared medical appointments based on the clinic's needs. Format 15 min: Check-in, vital signs, complete behavior questions, concerns 60 min: Interactive education and medical evaluation 15 min: Goal setting, check out	37	≥ 18	Four months	Exercise	A quantifiable goal was set by each participant, and 97% of them said they had either reached or almost reached their objective. Compared to women, men reported considerably more time spent exercising.
Fort et al,^16^ 2015	USA	The usual care group patients received a clinic visit with their primary care physician and laboratory tests. In Costa Rica, the standard care for patients with diabetes is every three months, and for hypertension, it is every four months. Patients receive their medications on the day of their clinic visit. For Chiapas, patients with diabetes and hypertension were seen every month, and at the same monthly visit, they had relevant lab tests done and picked up their medication.	75	≥ 21	Eight months	1) Self-efficacy measure 2) Stages of change measure 3) Diet index 4) Meets Recommended minutes of PA per week (%)	Interventions including group education at health facilities may enhance clinical results in addition to enhancing stage-of-change activation.
Gerage et al,^26^ 2020	Brazil	Control Group participants attended an educative lecture about lifestyle changes, whereas those in the VAMOS Group took part in a 12-week behavioral change program	90	≥ 40	Three months	PA, EH	The program improved blood pressure and microvascular reactivity in patients with hypertension.
Gerage et al,^27^ 2017	Brazil	Control Group participants attended an educative lecture about lifestyle changes, whereas those in the VAMOS Group took part in a 12-week behavioral change program	90	≥ 40	Three months	1) PA and EH; 2) Fruit (portions per day) 3) Water consumption 4) Salt consumption (g) 5) sugar consumption 6) Oil consumption 7) Self-efficacy for PA 8) Self-efficacy for EH	Patients with hypertension saw improvements in their EH and overall quality of life after using the VAMOS program.
Hyman et al,^17^ 2007	USA	(1) Simultaneous behavioral interventions, in which participants received information and counseling regarding all three target behaviors simultaneously for 18 months. (2) Sequential behavioral interventions, in which the target behaviors were introduced one at a time at 6-month intervals. The order in which the behaviors were introduced to each participant was randomized to avoid confounding outcomes with patient preferences. (3) Usual care was provided with a brief review of educational materials regarding the three target behaviors, with no telephone follow-up.	289	45-64	6 and 18 months	1) Smoking cessation 2) Sodium reduction 3) Increased PA	While both the simultaneous and sequential groups showed some effectiveness in achieving single behavioral goals, the simultaneous group had better outcomes at the 6-month mark for reducing dietary sodium. Overall, the study concluded that long-term multiple behavior change is challenging in primary care and that simultaneous counseling may be more effective than a sequential approach.
Jahan et al,^30^ 2020	Japan Bangladesh	The intervention group received five months of in-person health education along with a health education booklet and SMS text messaging to develop awareness and knowledge and motivate them for behavior changes, with the content of both educational materials and SMS text messaging being the same.	420	≥ 18	Five months	1) Salt intake 2) Fruits intake 3) Vegetable intake 4) PA 5) Blood pressure monitoring 6) Body weight monitoring	To maximize the efficacy of face-to-face health education, home health care services must be integrated with more pertinent and timely interactive SMS text messages.
Jalali et al,^24^ 2022	Iran	The individuals in the intervention group were given multimedia training software designed based on TTM and a phone number to call if they had any questions.	120	30-50	Six months	1) PA (min/wk) 2) Self-efficacy	Using multimedia educational interventions that are based on TTM might be one of the most effective ways to encourage PA and help individuals prevent hypertension.
Johnson et al,^18^ 2006	USA	The Pro-Change Program for High Blood Pressure Medication, based on the TTM, is a computer-generated, individualized, stage-matched expert system intervention and stage-based manual for adherence to antihypertensives.	1227	18-80	6,12,18 months	Prescribed medication, adherence, health care utilization	Regardless of their willingness to adapt, whole groups of noncompliant people may be significantly impacted by TTM-based expert system intervention.
Karupaiah et al,^31^ 2015	Malaysia	Patients received the lifestyle modification program provided by dieticians.Three healthy lifestyle behaviors related to blood pressure control were identified for dietician moderation, namely, reducing salt intake, regular exercise, and increasing fruit and vegetable intakes	302	≥ 18	Six months	1) Sodium intake (mg) 2) Pedometer steps (counts per day) 3) Total activity (MET, min/wk) 4) Fruits and vegetable intake (servings)	While self-reported adherence to recommended behaviors was low, clinically significant improvements were observed in systolic and DBP, weight, and waist circumference among those who adhered to sodium reduction and increased fruit and vegetable intake.
Kassavou et al,^28^ 2020	United Kingdom	Intervention group patients received highly tailored and interactive text and voice recognition messages; the intervention development was guided by the theoretical framework and included behavior change techniques and strategies mapped onto either or both intentional and nonintentional non-adherence	135	≥ 18	12 weeks	Medication adherence	Results showed significant improvements in medication adherence for the intervention group compared to the control group, alongside modest reductions in SBP and hemoglobin A1c levels. The intervention demonstrated high fidelity, engagement, and satisfaction, indicating its potential for clinical effectiveness in primary care settings
Liu et al,^32^ 2020	Canada	The e-Counseling and Control interventions were organized into 28 self-guided e-sessions (28 sessions in total), and they were delivered to the participants via email on a set schedule.	264	35-74	4 and 12 months	Daily steps Fruits and vegetable intake sodium intake restriction	The study found that patients in the e-Counseling group significantly increased their daily steps compared to the control group at the 12-month follow-up, and female participants in the e-Counseling group also showed a reduction in urinary sodium levels. Improvements in physical activity and dietary sodium intake were linked to reductions in blood pressure and Framingham Risk Index scores at the same follow-up.
Meuleman et al,^34^ 2017	Netherlands	1-hour individual motivational interview	138	≥ 18	Six months	Reduction of sodium intake	Participants in the intervention group experienced significant reductions in sodium excretion, blood pressure, and protein excretion, along with improved self-efficacy compared to the regular care group. However, by the six-month follow-up, while some benefits in office blood pressure and protein excretion persisted, differences in sodium excretion and ambulatory blood pressure were no longer significant
Motlagh et al,^9^ 2017	Iran	Participants attended a four-session training program in which meetings were delivered weekly.	78	≤ 69 years	n/a	Adherence to PA	A theory-based training intervention significantly increased physical activity and reduced 24-hour ambulatory blood pressure in hypertensive patients. The experimental group showed an increase of metabolic equivalents in physical activity compared to the control group, along with significant reductions in systolic and DBP. Additionally, a higher percentage of participants in the experimental group progressed to the action stage of exercise, with improvements in exercise self-efficacy and decisional balance.
Naeemi et al,^25^ 2022	Iran	For the experimental group, three 60-minute training sessions with a maximum capacity of 15 people were held.	99	≥ 60	Three months	Self-care	An educational intervention based on the health belief model significantly improved self-care behaviors and awareness related to hypertension among elderly participants after three months. Key constructs such as perceived sensitivity, perceived severity, perceived benefits, and action guidance showed marked increases in the experimental group compared to the control group.
Riches et al,^35^ 2021	United Kingdom	The Salt Swap intervention aimed to reduce dietary salt intake by encouraging individuals to swap to lower-salt alternatives when grocery shopping, buy fewer high-salt foods, and use less salt when cooking or at the table	47	≥ 18	Not specified	Reduction of dietary salt intake	The feasibility study involved 47 participants and successfully met all progression criteria, including a high follow-up attendance rate (96%) and strong intervention fidelity (81%). While the intervention did not significantly reduce salt intake, the salt content of purchased foods, or blood pressure, it demonstrated acceptability and feasibility within primary care settings, positively influencing participants' salt intake behaviors.
Piette et al,^19^ 2012	USA	Intervention patients completed 379 automated calls during 565 patient-call weeks, yielding an average call completion rate of 67%. At follow-up, intervention patients reported a variety of responses to information provided during the calls, including taking medication more regularly (70%), dietary changes (70%), and talking with their doctor about hypertension (61%).	200	18-80	6-12 months	Medication adherence	intervention patients experienced a 4.2 mm Hg reduction in SBP compared to controls, with a significant 8.8 mm Hg decrease in a subgroup with high information needs. Additionally, intervention participants reported fewer depressive symptoms, medication-related problems, better general health, and greater satisfaction with care
Rodriguez et al,^20^ 2021	USA	A telephone-delivered, behavioral stage-matched intervention, or a non-tailored health education intervention	533	Mostly ≥ 60	12 months	Diet. medication adherence, physical activity	Compared to usual care, participants in the stage-matched intervention group had an 84% higher likelihood of achieving BP control and a SBP reduction of 2.80 mm Hg, while the health education intervention group showed a 48% higher likelihood of control and a reduction of 2.58 mm Hg.
Wingo et al,^21^ 2013	USA	The established and established plus DASH interventions were provided in parallel series of group sessions, with 14 sessions during the initial six months supplemented with four individual intervention sessions.	537	≥ 18		Weight loss	While both dietary self-efficacy and exercise self-efficacy showed a significant negative relationship with weight change at 6 and 18 months, indicating that lower self-efficacy was associated with greater weight loss, changes in self-efficacy did not lead to expected behavior changes.
Young et al,^22^ 2010	USA	The “established” intervention promoted four lifestyle recommendations. The “established plus DASH” intervention included the same established guidelines plus the DASH dietary pattern. The advice-only comparison condition received verbal advice and materials on lifestyle modifications	762	≥ 25	6- and 18-months subscales	Increased physical activity, reduced daily sodium intake, reduced daily total fat intake	the established guidelines for blood pressure control (EST) improved three HRQOL subscales at 6 months and one at 18 months compared to the advice-only group, while the combination of guidelines and the DASH dietary pattern (EST + DASH) improved two subscales at both time points. Additionally, improvements in dietary intake and achieving a weight loss of at least 4 kg were associated with enhancements in HRQOL, underscoring the importance of dietary changes and weight management in promoting well-being.
Zhao et al,^23^ 2021	USA	Telephone delivered, transtheoretical stage-matched intervention based on the TTM	533	Mostly ≥ 60	Six-month intervention followed by a six-month observation	1. Diet self-efficacy. 2. Exercise self-efficacy 3. Medication self-efficacy	The study established that the self-report measure for self-efficacy in hypertension treatment adherence is both reliable and valid for adults with uncontrolled hypertension. The instrument demonstrated good internal consistency, with Cronbach's α values of 0.81 for diet self-efficacy, 0.82 for exercise self-efficacy, and 0.74 for medication self-efficacy, and exploratory factor analysis confirmed its three distinct subscales.
Chen et al,^33^ 2022	China	Patients in the intervention group received TTM-based health intervention, and those in the control group received usual care.	400	≥ 18	12 months	Medication adherence	The study found that a TTM-based health intervention significantly reduced SBP by 4.534 mm Hg, 3.982 mm Hg, and 5.803 mm Hg at 3, 6, and 12 months, respectively, and DBP by 3.383 mm Hg and 3.129 mm Hg at 3 and 12 months. Additionally, medication adherence improved significantly at all follow-up points, with patients in the intervention group showing an increased likelihood of being in a higher stage of medication adherence.

Abbreviations: DASH, Dietary Approaches to Stop Hypertension; EH, Eating habit; PA, Physical activity; TTM: Transtheoretical model

**Table 2:** The quality of quasi-experimental studies.

**Study**	**Q1**	**Q2**	**Q3**	**Q4**	**Q5**	**Q6**	**Q7**	**Q8**	**Q9**	**Overall quality **
Daley et al^14^	Y	Y	Y	N	N	Y	Y	Y	Y	Moderate
Dickman et al^15^	Y	N	Y	N	U	Y	Y	Y	Y	Moderate
Fort et al^16^	Y	Y	Y	N	N	Y	Y	Y	Y	Moderate
Jalali et al^24^	Y	Y	Y	Y	N	Y	Y	Y	Y	High
Karupaiah et al^31^	Y	Y	Y	N	U	Y	Y	Y	Y	Moderate
Naeemi et al^25^	Y	Y	Y	Y	U	Y	Y	Y	Y	High

Abbreviations: Q: Question; Y: Yes; N: No; U: Unclear.

## Discussion

 The current systematic review demonstrated the importance of behavior change interventions, including dietary modifications and increased physical activity, in managing hypertension and improving overall health outcomes. Tailored approaches based on individual characteristics such as gender may enhance the effectiveness of lifestyle interventions.

 Hypertension is a significant risk factor for heart disease and stroke, two of the leading causes of death in the United States.^36^ Effective treatment and management of hypertension can improve quality of life, but effective treatment requires patient cooperation and self-management. Lifestyle modifications, such as changes in diet, are crucial for achieving better results. It is important to note that managing hypertension in elderly patients requires special consideration.^37^ Most of the studies that have been reviewed included senior participants. This highlights the need for changes in interventions delivered to older people, as not all of them can access the internet or have feasible telephone calls, and in-clinic visits are also not easily attainable. Therefore, healthcare professionals must consider these factors in every procedure and intervention designed for elderly patients. As it is predicted that more than 70% of medical practice will be directed toward geriatric needs in the coming years, managing hypertension in the elderly is an issue of great importance for clinicians.

 Hypertension is often referred to as the “silent killer” because it can go unnoticed in individuals with high blood pressure. Symptoms may not be visible until a significant event, such as a stroke, occurs, which may cause hypertensive patients to underestimate the severity of their condition.^38^ Overall, studies show that hypertension and awareness of it are associated with lower quality of life scores due to the adverse effects of the anti-hypertensive medications used in the treatment and chronic characteristics of the disease.^39^ For example, adverse reactions in the skin, like eczema, have been observed as an adverse event.^40^

 TTM is a valuable strategy for promoting behavior change and improving lifestyle. Precontemplation, contemplation, preparation, action, maintenance, and termination are the six phases of change that the TTM suggests one must go through in order to modify one’s behavior for health.^41^ TTM has been used for inducing different behavioral change applications including diet and exercise since its introduction.^42^ Research has shown that TTM-based interventions can be effective in promoting behavior change across a range of health behaviors, especially in smoking behavior. For instance, in a study conducted by Martinasek et al^43^ predictors of the TTM were assessed in college-aged vaping students and found that compared to their male counterparts, females tended to be further along in the stages of transformation. Furthermore, compared to younger students, older students were more likely to be in the maintenance stage. Students who vaped for a longer period of time tended not to have advanced into any other stage of change except contemplation.

 In the hypertension context, the application of TTM shows that it can be effective in promoting behavior change related to management, such as medication adherence and lifestyle modifications.^44^ Another cross-sectional survey of 299 hypertensive participants^45^ indicated that the TTM’s cognitive aspect is an independent predictor of physical activity behavior and may play a significant role in physical activity behavior change in patients with hypertension.

 The current study reviewed various types of studies, including randomized controlled trials, quasi-experimental studies, and other interventional studies to investigate the effectiveness of the TTM model in helping hypertensive patients in most of the studies. Behavior changes, including increasing physical activity, diet changes, medication adherence, etc, have happened, which can quickly and directly influence blood pressure levels and help patients in controlling blood pressure levels. Behavior changes Most studies proved that the intervention, like education delivered in a transtheoretical framework, has resulted in positive modifications. For example, it is supported by solid pieces of evidence that increased physical activity is associated with lower and controlled blood pressure levels,^46,47^ and in 16 out of 23 reviewed studies, increased level of physical activity and exercise was demonstrated as a result of the educational stage of change model.

 Likewise, for older adults, further studies are required to be conducted in children. Karami Daranjani et al investigated the TTM model in hypertensive children and indicated that it promotes the physical activity of children patients,^48^ but more studies are needed to address the issue.

 Inadequate adherence to treatment is another barrier to effective treatment. Six included studies had investigated this condition and had recorded positive changes using the behavior of change model. In general, it was demonstrated that TTM-based interventions are potentially significant for patients who fail to adhere, regardless of their readiness to change.

 On the other hand, several studies included in the current systematic review pointed out the role and significance of self-efficacy in behavioral modifications. For example, changes in self-efficacy may occur following individuals’ active and successful participation in physical activity. Moreover, self-efficacy has a predictability power of self-care behavior in chronic diseases such as diabetic patients.^49^

 The systematic review by Hasriani et al investigated 5 papers and suggested TTM-based educational intervention can lead to a low-salt diet and behavioral modification in hypertensive individuals; these behavioral changes influence clinical changes like reduced urine salt excretion, reduced weight and waist circumference, reduced blood pressure, and reduced risk of cardiovascular disease.^50^

 Another systematic review in 2022^51^ investigated the use of the TTM in medication adherence, suggesting that TTM-based interventions in patients with low or moderate medication adherence are effective, but more studies are needed. The TTM model was also used for psychoeducation, indicating that it can be effective in schizophrenia treatment by making positive lifestyle changes. It can be concluded that TTM application cannot only be practical in chronic conditions like hypertension and diabetes mellitus, as shown in previous studies, but it can also be effective in treating diseases like schizophrenia.

## Strengths and limitations

 The current systematic review demonstrates a robust methodology, including clear protocol and registration, comprehensive search strategy, rigorous study selection, and data extraction process, and conducting of risk of bias assessment in the included studies. However, it was also the subject of several limitations which are discussed in the further paragraphs.

 The foremost limitation was the measure outcomes were evaluated with, originating from the fact that the nature of outcomes did not fully allow quantified assessment. When assessing HRQOL, although the impact of hypertension on HRQOL has been investigated, determining what exactly quality of life means in this context is a complex task that has yet to be resolved. There is currently no consensus on how to define and quantify the quality of life for hypertensive patients, and no single survey tool has been established as the gold standard for assessing this critical aspect of their well-being. The Symptom Rating Test,^52^ the Sickness Impact Profile,^53^ and the 36-Item Short Form Survey^54^ are some examples of existing tools. The three reviewed articles which have assessed HRQOL, have used the general question of the World Health Organization Quality of Life questionnaire, brief version,^27^ The Rand 36-item Health Survey,^22,55^ and the 5-level EQ-5D.^28,56^ In addition, the heterogeneous nature of the interventions, which was comprehensively described in the previous sections, restricts the generalizability of the findings of the current study.

 Another limitation is the relatively high risk of bias, especially performance and detection bias. Reducing bias can be challenging due to the type of intervention delivered and the outcome assessment process. When interventions are delivered through phone calls, in-clinic visits, lectures, and so on, blinding becomes difficult to achieve, which can significantly impact the results. Another challenge in this review was the difficulty of quantification and mixing the results, as the outcomes and the measurements were dissimilar. Lastly, the duration of the follow-up was too short to address the long-term efficacy of the interventions.

 Further research is required to investigate different aspects of this topic thoroughly. Additional studies should be conducted in various healthcare settings and populations. It is important to note that although most reviewed studies involved participants being revisited in several follow-ups, a long-term follow-up is necessary to assess outcomes such as cardiovascular events and mortality. Future investigations can also explore the implementation of technology-based interventions such as telemedicine.

## Conclusion

 The type and duration of interventions varied among the studies reviewed, with the shortest duration being three months, indicating the time-consuming nature of the treatment. Several studies also included multiple follow-ups and assessments after the intervention highlighting the association between behavior change and time. It is important to note that habit formation requires ample time, but there may be barriers to maintaining lifestyle modifications over an extended period. Overall investigation indicates that the TTM and stage of change model can be an appropriate framework to deliver educational messages to patients and have them change behavior and lifestyle modifications to effectively control blood pressure levels.

## Competing Interests

 Authors declare no competing interests.

## Ethical Approval

 The current study was conducted on previously published materials with no direct patient communication.

## Supplementary Files


Supplementary file 1 presents the Checklist for Quasi-Experimental Studies (Non-Randomized Experimental Studies).
